# A Phos-Tag-Based Approach Reveals the Extent of Physiological Endoplasmic Reticulum Stress

**DOI:** 10.1371/journal.pone.0011621

**Published:** 2010-07-16

**Authors:** Liu Yang, Zhen Xue, Yin He, Shengyi Sun, Hui Chen, Ling Qi

**Affiliations:** 1 Graduate Program in Biochemistry, Molecular and Cell Biology, Cornell University, Ithaca, New York, United States of America; 2 Graduate Program in Nutrition, Cornell University, Ithaca, New York, United States of America; 3 Graduate Program in Genetics and Development, Cornell University, Ithaca, New York, United States of America; 4 Division of Nutritional Sciences, Cornell University, Ithaca, New York, United States of America; Johns Hopkins School of Medicine, United States of America

## Abstract

Cellular response to endoplasmic reticulum (ER) stress or unfolded protein response (UPR) is a key defense mechanism associated with many human diseases. Despite its basic and clinical importance, the extent of ER stress inflicted by physiological and pathophysiological conditions remains difficult to quantitate, posing a huge obstacle that has hindered our further understanding of physiological UPR and its future therapeutic potential. Here we have optimized a Phos-tag-based system to detect the activation status of two proximal UPR sensors at the ER membrane. This method allowed for a quantitative assessment of the level of stress in the ER. Our data revealed quantitatively the extent of tissue-specific basal ER stress as well as ER stress caused by the accumulation of misfolded proteins and the fasting-refeeding cycle. Our study may pave the foundation for future studies on physiological UPR, aid in the diagnosis of ER-associated diseases and improve and facilitate therapeutic strategies targeting UPR *in vivo*.

## Introduction

ER homeostasis is tightly monitored by ER-to-nucleus signaling cascades termed UPR [1]. Recent studies have linked ER stress and UPR activation to many human diseases including heart complications, neurodegenerative disorders, and metabolic syndrome [1], [2]. Indeed, chemical chaperones and antioxidants aiming to reduce ER stress and UPR activation have been shown to be effective in mouse models of obesity and type-1 diabetes [3]–[5]. Despite recent advances, our understanding of UPR activation under physiological conditions is still at its infancy, largely due to the lack of sensitive experimental systems that can detect mild UPR sensor activation.

The underlying mechanisms of UPR signaling and activation induced by chemical drugs such as thapsigargin (Tg) are becoming increasingly well-characterized [1]. Upon ER stress, two key ER-resident transmembrane sensors, inositol-requiring enzyme 1 (IRE1α) and PKR-like ER-kinase (PERK) undergo dimerization or oligomerization and trans-autophosphorylation via their C-terminal kinase domains, leading to their activation [1], [2]. Phosphorylation of IRE1α and PERK has been challenging, if not impossible, to detect under physiological conditions. The mobility-shift of IRE1α shown in many studies is very subtle and, as demonstrated in this study, may be inaccurate and misleading. In addition, commercially-available phospho-specific antibodies (e.g. P-Ser724A IRE1α and P-Thr980 PERK) do not reflect the overall phosphorylation status of the proteins. Finally, use of these antibodies, if successful, raises the question as to whether Ser724 of IRE1α or Thr980 of PERK is indeed phosphorylated under various physiological and disease conditions.

Alternatively, many studies have used downstream effectors such as X-box binding protein 1 (XBP1) mRNA splicing, phosphorylation of eukaryotic translation initiation factor 2a (eIF2α), C/EBP homologous protein (CHOP) and various genes involved in protein folding and ER-associated degradation (ERAD) as surrogate markers for UPR activation. This method, albeit convenient, may be confounded by the possibility of integrating signals not directly related to stress in the ER. For example, the PERK pathway of the UPR is part of the integrated stress response that consists of three other eIF2α kinases [1]. Activation of any of these kinases leads to eIF2α phosphorylation and induction of ATF4 and CHOP [1]. A recent study also showed that ATF4 and CHOP can be regulated translationally in a PERK-independent manner via the TLR signaling pathways [6]. Furthermore, UPR target genes such as CHOP and ER chaperones can be induced by other signals, such as insulin and cytokines/growth factors [7], [8]. Thus, downstream UPR targets alone are not best suited for accurate assessment and evaluation of UPR status, especially under physiological and disease settings.

Our previous study utilized the Phos-tag-based system [9] to detect IRE1α phosphorylation mainly in Tg-treated culture cells [10]. Here we have further modified the system to maximize the resolution of IRE1α phosphorylation and extended the system to detect PERK phosphorylation. Strikingly, our system allows for increased sensitivity in detecting UPR activation and more importantly, accurate quantitation of ER stress. This powerful tool allows us to quantitatively measure the extent of UPR or ER stress induced by various physiological conditions, including (a) the accumulation of misfolded proteins in HEK293T cells, (b) the basal feeding conditions in various adult tissues and (c) the fasting-feeding cycle in the pancreas. Our data reveal that many tissues and cell types constitutively display mild ER stress and more intriguingly, various acute physiological challenges increase ER stress by 2–3 fold over basal levels.

## Results

### Visualization of sensor phosphorylation and quantitation of ER stress

**Figure 1:**
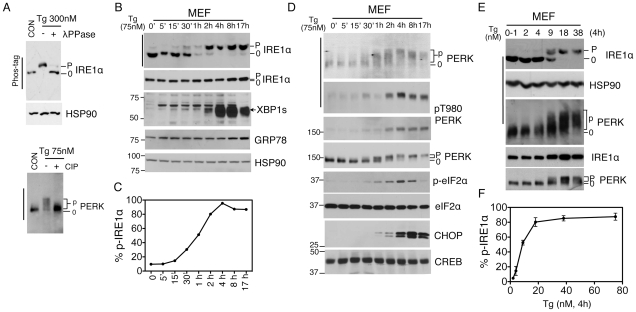
Visualization and quantitation of ER stress under pharmacological stress. (**A**) Immunoblots of IRE1α (upper) and PERK (lower) proteins in Tg-treated MEFs treated with or without λPPase or CIP. (**B and D**) Immunoblots of IRE1α (**B**) and PERK (**D**) using the Phos-tag vs. regular gels. MEFs were treated with 75 nM Tg at indicated period of time. (**C**) Quantitation of percent of phosphorylated IRE1α in total IRE1α protein in Phos-tag gels shown in **B**. (**E**) Immunoblots of IRE1α and PERK in wildtype MEFs treated with Tg at indicated concentrations for 4 h. (**F**) Quantitation of percent of phosphorylated IRE1α in total IRE1α protein in Phos-tag gels in **E**. HSP90 and CREB, loading controls. Phos-tag gels are indicated with a bar at the left-hand side. “0” refers to the non- or hypophosphorylated forms of the protein whereas “p” refers to the phosphorylated forms of the protein.

We optimized the separation of phosphorylated IRE1α and PERK proteins in a Phos-tag-based Western blot (see Methods section and Figure S1), which was reversed by phosphatase treatment (Figure 1A). Strikingly, IRE1α and PERK hyperphosphorylation patterns were distinct (Figure 1A), reflecting various levels of phosphorylation upon activation. Dramatically, p-IRE1α exhibited one discrete slow–migrating band in the Phos-tag gels, a feature that allows for quantitation of the percent of p-IRE1α (see below). Upon treatment with Tg, the percent of phosphorylated IRE1α increased from 30 min post-treatment, peaked around 4 h and slightly decreased at 8–17 h, with nearly 30, 100 and 80% of IRE1α undergoing phosphorylation, respectively (Figure 1B–C). Similarly, PERK hyperphosphorylation increased at 30 min, peaked at 4 h and decreased after 8–17 h. In both cases, the dynamic patterns of IRE1α and PERK phosphorylation were either not discernible or less impressive in regular gels or using the phospho-specific antibody (Figure 1B and D).

The temporal dynamic patterns of IRE1α and PERK phosphorylation as shown above indicate that hyperphosphorylation of UPR sensors correlates with the amount of stress in the ER. Further supporting this notion, hyperphosphorylation of IRE1α and PERK increased with Tg concentrations, peaking and subsequently plateauing at 38 nM Tg upon 4 h treatment (Figure 1E). Demonstrating the sensitivity and quantitative nature of our method, ∼15% of IRE1α protein were phosphorylated upon 4 nM Tg treatment and increased to ∼50% under 9 nM Tg (Figure 1E–F). In contrast, IRE1α phosphorylation was not visible using a regular gel system and phosphorylation of PERK was also much less impressive (Figure 1E). Thus, our method achieves both accuracy and sensitivity in detecting ER stress and UPR activation. We then went on to characterize the extent of ER stress under three physiological conditions.

### Accumulation of misfolded proteins induces mild ER stress

**Figure 2:**
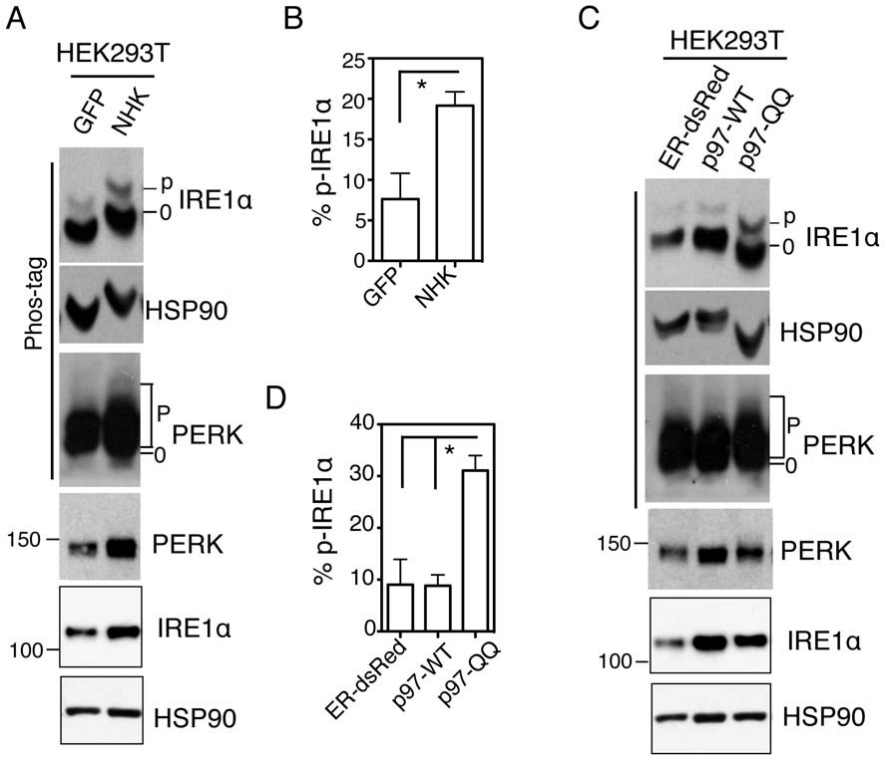
Accumulation of misfolded proteins induces mild ER stress. (**A and C**) Immunoblots of IRE1α and PERK in HEK293T cells transfected with the indicated plasmids for 24 h. NHK, the unfolded form of α1-antitrypsin; p97-QQ, dominant negative form of p97-WT. ER-dsRed and GFP, negative control plasmids. HSP90, a position and loading control. (**B and D**) Quantitation of percent of phosphorylated IRE1α in total IRE1α protein in Phos-tag gels shown in **A**, **C**. Values are mean ± SEM *, *P*<0.05 using unpaired two-tailed Student's *t*-test. Representative data from at least three independent experiments shown.

Although ER stress was initially characterized as induced by accumulation of unfolded proteins [11]–[13], it remains impossible to quantitate the levels of stress inflicted by accumulation of misfolded proteins in the ER. To this end, we ectopically expressed the terminally-misfolded α1-antitrypsin (AT) genetic variant-null Hong Kong (NHK) (Figure 2A), a frequently mutated allele in human α1 AT deficiency [14] or the dominant-negative mutant of p97 (p97-QQ) (Figure 2C), a member of the AAA-ATPase protein family involved in ERAD [15]. In both cases, IRE1α and PERK were phosphorylated when compared to cells overexpressing control or wildtype proteins (Figure 2A and C), indicating the specificity of sensor activation in response to misfolded proteins. Interestingly, IRE1α phosphorylation nearly tripled in both cases reaching 20–30% (Figure 2B–D). Similar observations were obtained in Sel1l-deficient MEFs (not shown), in which ERAD is defective [16]. Thus, our data revealed quantitatively the extent of ER stress induced by accumulation of misfolded proteins in the ER, a finding that was impossible using regular systems under similar running conditions (Figure 2A and C).

### Many tissues exhibit basal ER stress under feeding conditions

**Figure 3:**
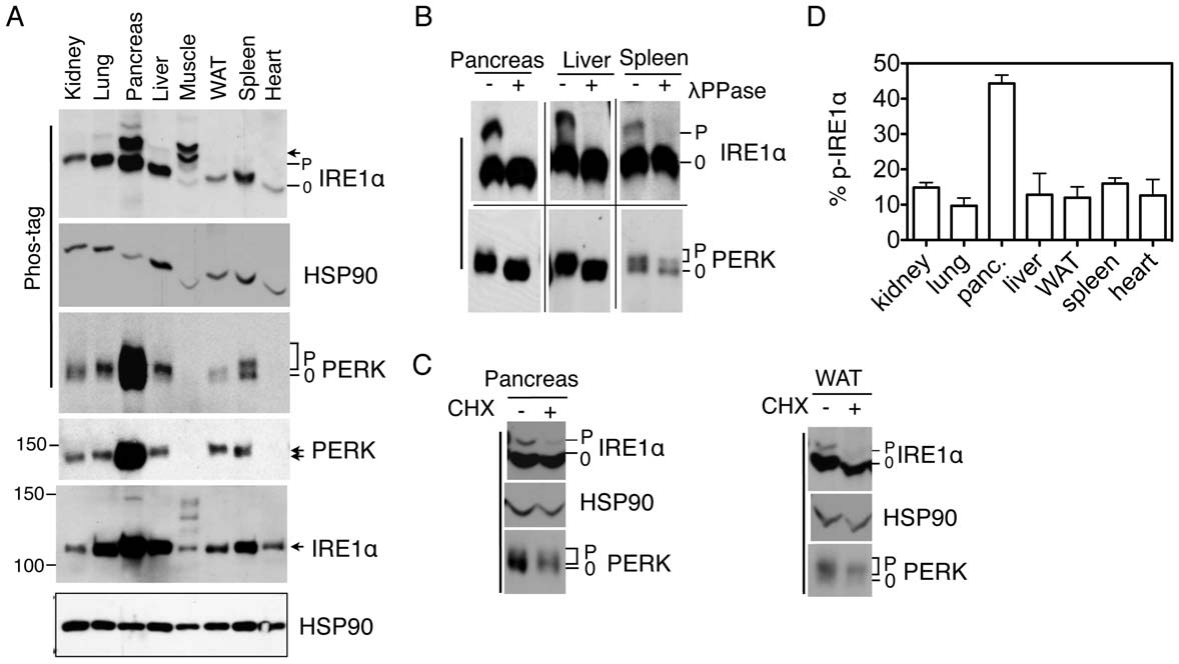
Many tissues exhibit basal ER stress under feeding conditions. (A) Immunoblots of IRE1α and PERK in various tissues of wildtype mice. WAT, white adipose tissues; Panc, pancreas; Muscle, gastrocnemius. HSP90, a position and loading control. (**B–C**) Immunoblots of IRE1α and PERK in tissue lysates treated with λPPase (**B**) or in pancreatic and WAT lysates prepared from mice injected with CHX (**C**). (**D**) Quantitation of percent of phosphorylated IRE1α in total IRE1α protein in various tissues shown in A. Values are mean ± SEM. Representatives of at least two independent experiments shown.

We then analyzed the levels of basal ER stress in various tissues from adult mice under feeding conditions. Intriguingly, many tissues exhibited slower electrophoretic mobility of IRE1α and PERK proteins (Figure 3A and S2A). The mobility shift of IRE1α and PERK was specific for phosphorylation as it was reversed by phosphatase treatment (Figure 3B and S2B); importantly, this was caused by signals from the ER as it was attenuated in the presence of a protein translation inhibitor, cycloheximide (CHX) (Figure 3C). Quantitatively, phosphorylated IRE1α accounted for over 40% of total IRE1α protein in the pancreas and ∼10% in most of the other tissues (Figure 3D). Our data is in line with an early finding in which the XBP1-GFP reporter mice exhibited basal UPR primarily in the pancreas [17]. Pointing to the complexity of tissue-specific UPR, IRE1α exhibited multiple slower migrating bands and PERK was beyond the detection limit in skeletal muscle (Figure 3A and S2A). The nature of these slower migrating bands in the IRE1α blot was not due to phosphorylation as they were resistant to phosphatase treatment (Figure S2C).

### Refeeding induces mild ER stress in the pancreas

**Figure 4:**
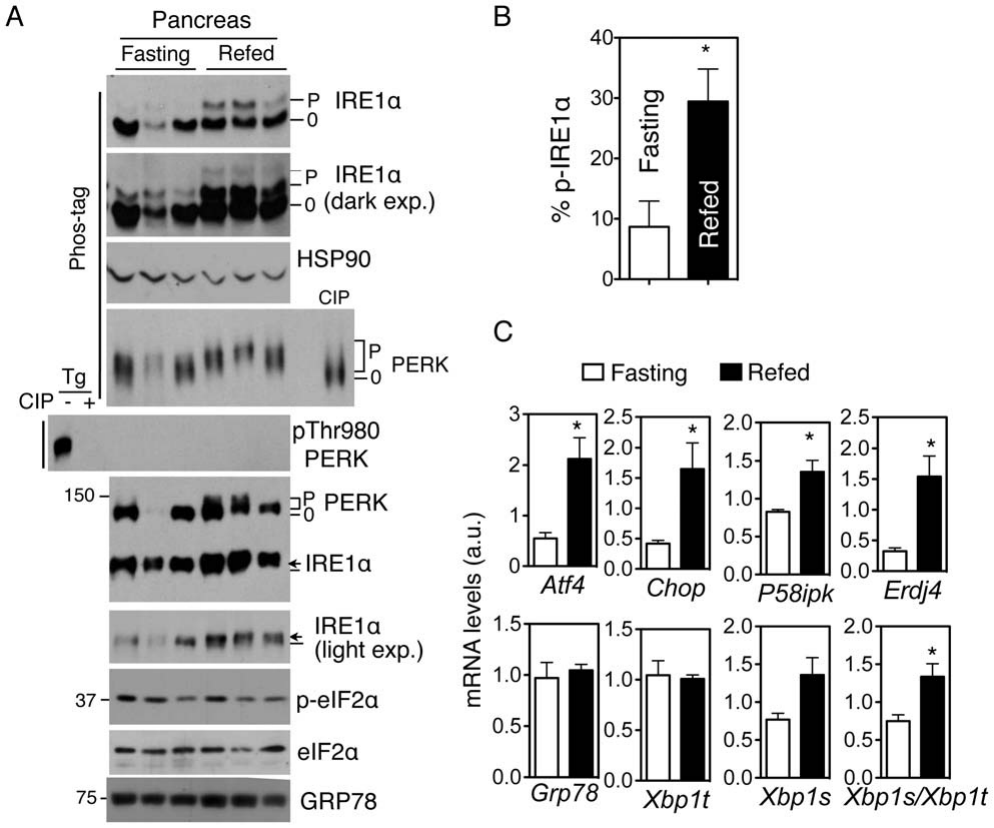
Fasting-refeeding induces mild ER stress in pancreas. (**A**) Immunoblots of lysates from the pancreas of wildtype mice either fasted or fasted followed by 2 h refeeding (refed). For the PERK blot, a mixture of all 6 samples treated with CIP were included as a control. For the p-PERK blot, Tg-treated MEF cell lysates with or without CIP treatment were included as a control. HSP90, a loading control. (**B**) Quantitation of the percent of phosphorylated IRE1α in pancreas under fasting and refeeding conditions shown in A (N = 4 mice per cohort). (**C**) Q-PCR analyses of UPR genes in the pancreas under either fasting or refeeding. Values are mean ± SEM. *Xbp1t*, total *Xbp1*; *Xbp1s/Xbp1t*, splicing efficiency. N = 3–4 mice. *, *P*<0.05 using unpaired two-tailed Student's *t*-test. Representatives of at least two independent experiments shown.

We then conducted an in-depth analysis of UPR activation during the fasting-refeeding process in the pancreas (20 hr fasting followed by 2 hr feeding). Indeed, refeeding significantly increased phosphorylation of both IRE1α and PERK (percent of p-IRE1α under fasting vs. refeeding: 8.7±4.3% vs. 29.5±5.4%; P<0.05) (Figure 4A–B). This effect was independent of the region of the pancreas sampled (Figure S2D). Supporting the importance of our method in analyzing mild physiological UPR, similar running conditions in regular gels resulted in a much less impressive mobility-shift for PERK (Figure 4A). This mild PERK phosphorylation was undetectable using the phospho-PERK antibody (Figure 4A). In addition, although IRE1α did exhibit a slightly slower mobility shift upon refeeding in regular gels after prolonged gel running conditions, this shift did not reflect the overall phosphorylation status of IRE1α as revealed by the Phos-tag gel (Figure 4A). Furthermore, phosphorylation of eIF2α, an immediate downstream effector of PERK, did not change (Figure 4A). Finally, while some UPR targets such as CHOP, ERDJ4 and P58IPK were induced upon refeeding (Figure 4C), both the mRNA and protein levels of Grp78, an ER chaperone, were not altered (Figure 4A and C). Thus, our data demonstrated that the fasting-feeding cycle acutely stimulates mild UPR activation in the pancreas.

## Discussion

In summary, we have optimized a sensitive and simple Phos-tag-based system to quantitatively assess ER stress and UPR activation with the following major advantages: First, dynamic ranges of PERK and IRE1α phosphorylation can be more sensitively visualized compared to regular SDS-PAGE gels; this is particularly important for physiological UPR where ER stress can be so mild that traditional methods may no longer be accurate or reliable. Second, the major breakthrough of our method lies in the unique pattern of IRE1α phosphorylation in the Phos-tag gel, which allows for a quantitative assessment of ER stress. To our knowledge, this is the first demonstration of quantitation of ER stress under physiological or pathological settings (e.g. the fasting-refeeding cycle or the accumulation of misfolded proteins). Finally, in comparison to using commercially-available phospho-specific antibodies (e.g. P-Ser724A IRE1α and P-Thr980 PERK), our method not only provides a complete view of the overall phosphorylation status of IRE1α and PERK proteins, but also circumvents the issue of whether these specific residues are indeed phosphorylated under certain physiological conditions.

Our data reveal that many tissues and cell types display constitutive basal UPR activity, presumably to counter misfolded proteins passing through the ER. This observation is in line with an early report demonstrating that under physiological conditions removal of these misfolded proteins in yeast requires coordinated action of UPR and ERAD [18]. Taking it one step further, our data show that a fraction of mammalian IRE1α and PERK is constitutively active in many tissues, with ∼10% IRE1α being phosphorylated and activated. This low level of IRE1α activation and ER stress in many tissues may provide a plausible explanation for the inability of an earlier study to detect basal UPR in the XBP1s-GFP reporter mice [17]. We believe that this basal UPR activity, especially the IRE1α-XBP1 branch, is critical in maintaining ER homeostasis and providing quality control as supported by the embryonic lethality of IRE1α and XBP1-deficient mice [1], [19]–[22]. It is noteworthy that in skeletal muscles, IRE1α exhibited multiple non-phosphorylated bands while PERK protein is beyond the detection limit. As the IRE1α-XBP1 pathway is active in adult skeletal muscles [17], the role of UPR in myocytes is an interesting question as it may offer new insights into physiological UPR.

As exocrine pancreatic acinar cells account for over 80% of the pancreatic mass, pancreatic ER stress observed under the fasting-feeding cycle likely reflects the acute elevation of protein synthesis in acinar cells in response to food intake [23]. Indeed, mice with XBP1 or PERK deficiency exhibit defective development of exocrine pancreas [24]–[26], suggesting an indispensable role for UPR in countering the fluctuating stress associated with food intake. While UPR is mildly active under fasting presumably to attenuate protein synthesis as previously suggested [26], our data showed a 3-fold increase of IRE1α phosphorylation, i.e. UPR, to enhance ER homeostasis in preparation for an upcoming wave of protein synthesis. Our results are in line with earlier observations demonstrating that ER in pancreatic acinar cells becomes dilated within 2–4 h refeeding [27], [28]. Nonetheless, it is quite surprising that ER stress in pancreatic cells fluctuates with the fasting-feeding cycle because acute mild UPR would expectedly reset proteostasis upon each fasting-feeding cycle, leading to the expansion of the proteostasis network and adaptation [29]. Hence, we postulate that the proteostasis network in acinar cells is very flexible in order to respond to many variables in the feeding process. The same is likely to be true for pancreatic islet cells.

There are several potential applications for our method in both basic and clinical research. First, our method may help elucidate the activation mechanisms for IRE1α and PERK. The effect of critical residues or inter-/intra-molecular interactions on sensor activation as well as branch-specific activation of non-canonical UPR pathways can now be accurately measured and quantitated. Second, our method may aid in the diagnosis of UPR-associated diseases by providing a more sensitive tool for detecting ER stress. The knowledge of the extent of ER stress in a given tissue of a patient may help assess disease progression. Finally, our method may assist in drug development and design. The efficacy of drugs such as chemical chaperones or antioxidants on ER stress can be quantitatively measured based on sensor activation, circumventing the complications associated with crosstalk among various pathways.

As ER stress is being implicated in an increasing number of physiological processes as well as human diseases such as cancer, liver diseases, neurodegeneration and type-1 diabetes [1], [2], new strategies and approaches enabling a comprehensive understanding of UPR in physiological and disease settings are urgently needed to facilitate drug design targeting UPR in conformational diseases [2]. The ability to directly visualize and quantitate UPR activation is an important step towards gaining novel insights into physiological UPR and improving therapeutic strategies targeting UPR in vivo.

## Materials and Methods

### Cells and reagents

HEK293T and MEFs as described in [10] were maintained in DMEM supplemented with 10% FBS (Hyclone) and 1% penicillin/streptomycin. Tg (EMD Calbiochem) and stock CHX (Sigma) were dissolved in DMSO and ethanol, respectively. Cells were treated with Tg at indicated concentrations for the indicated times and immediately snap-frozen in liquid nitrogen. Phos-tag was purchased from NARD Institute (Japan).

### Protein lysates, Western blot and Phos-tag gels

Whole cell or nuclear extraction was performed as we previously described [10], [30]. Lysate protein concentrations were measured using the Bradford assay (Biorad) and normalized to 0.5∼2 µgµμl using SDS sample buffer. Samples were boiled for 5 min prior to loading onto a SDS-PAGE gel. 15–30 µg of whole cell lysates or 1–10 µg of nuclear extracts were used in a mini SDS-PAGE. Phos-tag gel was modified from our previous report [10] with the following running conditions: 100 V for 3 h for IRE1α using 25 µM Phos-tag and 15 mA for 15 min followed by 5 mA for 9.5 h for PERK using 3.5 µM Phos-tag. To achieve optimal results, we always run IRE1α and PERK on separate gels using the following conditions. Membranes were routinely strip-reprobed for 2–4 times. The IRE1α blot in the Phos-tag gel was routinely reprobed with HSP90 (90 kDa vs. 110 kDa IRE1α) as a position control.

Importantly, for both regular and phos-tag gels, gel-running was stopped when the 75 kDa maker ran off the gel and same amounts of lysates were loaded. Therefore, the difference in separating the phosphorylated from the non-phosphorylated species between Phos-tag and regular gels was mainly attributable to the effect of Phos-tag incorporated.

### Antibodies for Western blot

GRP78 (goat, 1∶1,000), XBP1 (XBP1u/s-specific, rabbit, 1∶1,000), CHOP (mouse, 1∶500) and HSP90 (rabbit, 1∶5,000) were purchased from Santa Cruz; p-eIF2α, eIF2α, IRE1α and (p)-PERK (rabbit) antibodies were purchased from Cell Signaling and used at 1∶1,000–2,000. Primary antibodies were diluted in 5% milk/TBST or 2% BSA/TBST and incubated with PVDF membrane overnight at 4°C. Secondary antibodies were goat anti-rabbit IgG HRP, goat anti-mouse IgG HRP (Biorad) and donkey anti-goat IgG HRP (Jackson ImmunoResearch), all of which were used at 1∶10,000.

### Mice and tissues

Wildtype C57BL/6 mice were purchased from the Jackson Laboratory or bred in our mouse facility. For some experiments, mice were injected with 40 µg CHX per g body weight (dissolved in 100 µl PBS) for 2 h. Epididymal white adipose tissues (WAT) and pancreas were harvested. Following cervical dislocation, tissues were harvested immediately, snap-frozen in liquid nitrogen and stored at −80°C. All animal procedures have been described previously [31], [32] and were approved by the Cornell IACUC (#2007-0051).

### Plasmids and transfection

NHK, wildtype and dominant negative E305Q/E578Q p97 (p97-QQ) plasmids were gifts from Qiaoming Long and Fenghua Hu (Cornell University), respectively. HEK293T were transfected with plasmids using polyethylenimine (PEI, Sigma) as we recently described [30]. Cells were snap-frozen in liquid nitrogen 24 h post-transfection followed by Western blot.

### Phosphatase treatment

100 µg cell lysates or tissue lysates were incubated with 2.5 µl calf intestinal phosphatase (CIP) or 0.5 µl lambda phosphatase (λPPase, New England BioLabs- NEB) in 1× NEB buffer 3 (100 mM NaCl, 50 mM Tris-HCl, 10 mM MgCl_2_, 1 mM DTT) or 1× PMP buffer (50 mM HEPES, 100 mM NaCl, 2 mM DTT, 0.01% Brij35, NEB) with 1 mM MnCl_2_ at 37 or 30°C for 45 or 30 min, respectively. Reaction was stopped by adding 5× SDS sample buffer and incubated at 90°C for 5 min.

### RNA extraction and Q-PCR

Total mRNA extractions were carried out using a combination of Trizol and RNeasy kit (Qiagen) for pancreas. RNAs were reverse transcribed using Superscript III kit (Invitrogen). For Q-PCR, cDNA were analyzed using the SYBR Green PCR system on the Roche 480 LightCycler (Roche). Reactions using samples with no RT and water were included as negative controls to ensure the specificity of the Q-PCR reaction. All Q-PCR data were normalized to ribosomal *l32* gene in the corresponding sample. Primer sequences are listed in Supplementary material Table S1.

### Image quantification

Quantification was performed using the NIH ImageJ software where band densities were calculated and subtracted from the background. Data are represented as mean ± SEM from several independent samples or experiments.

### Statistical analysis

Results are expressed as mean ± SEM. Comparisons between groups were made by unpaired two-tailed Student *t*-test. *P*<0.05 was considered as statistically significant. All experiments were repeated at least twice.

## Supporting Information

Figure S1 Immunoblots of p-Thr980 PERK, IRE1α (left) and total PERK (right) in different MEFs treated with or without Tg. (left) IRE1α−/− and PERK−/− MEFs were used; (right) wildtype (+/+), PERK−/− (−/−) and PERK−/− MEFs rescued with wildtype PERK (−/− + wt).
(0.16 MB JPG)

Figure S2 (A) Immunoblots of IRE1α (top) and PERK (bottom) in various tissues of wildtype mice under feeding conditions, an independent experiment from the one shown in Figure 3A. WAT, white adipose tissues; Panc, pancreas; Muscle, gastrocnemius. (B) Original Phos-tag whole-gel images for the data shown in Fig. 3B. Note the specificity of the antibody and the complete reverse of phosphorylation upon phosphatase treatment. (C) Immunoblots of IRE1α and PERK in muscle lysates treated with λPPase. The multiple bands of IRE1α in the muscle are not due to hyperphosphorylation and PERK protein levels are beyond detection limit. (D) Immunoblots of IRE1α and PERK in lysates extracted from different regions of the pancreas of 13-week-old wildtype mice under the 20 h-fasting (F) and 2 h-refeeding (R) conditions. The position of the pancreas is relative to the duodenum (proximal, middle or distal) - see the diagram on top. HSP90, a loading control. Phos-tag gels are indicated with a bar at the left-hand side.
(0.35 MB JPG)

Table S1 Primers used in this study.
(0.04 MB PDF)
