# Supplementary figures and images for: A Phos-Tag-Based Approach Reveals the Extent of Physiological Endoplasmic Reticulum Stress
Source: PLoS One. 2010 Jul 16;5(7):e11621. doi: 10.1371/journal.pone.0011621 (PMC2905412; doi:10.1371/journal.pone.0011621)

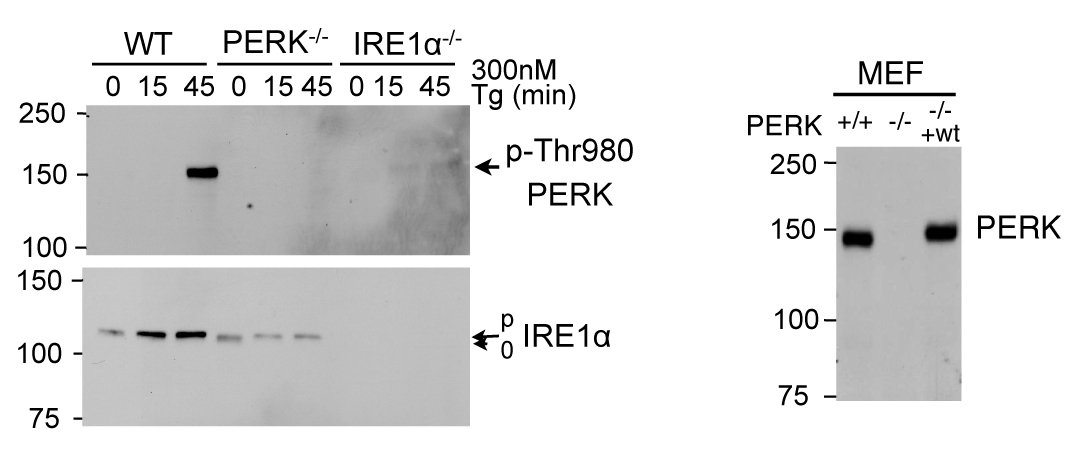

Supplement: Figure S1 — Immunoblots of p-Thr980 PERK, IRE1α (left) and total PERK (right) in different MEFs treated with or without Tg. (left) IRE1α−/− and PERK−/− MEFs were used; (right) wildtype (+/+), PERK−/− (−/−) and PERK−/− MEFs rescued with wildtype PERK (−/− + wt). (0.16 MB JPG) [file pone.0011621.s001.jpg]

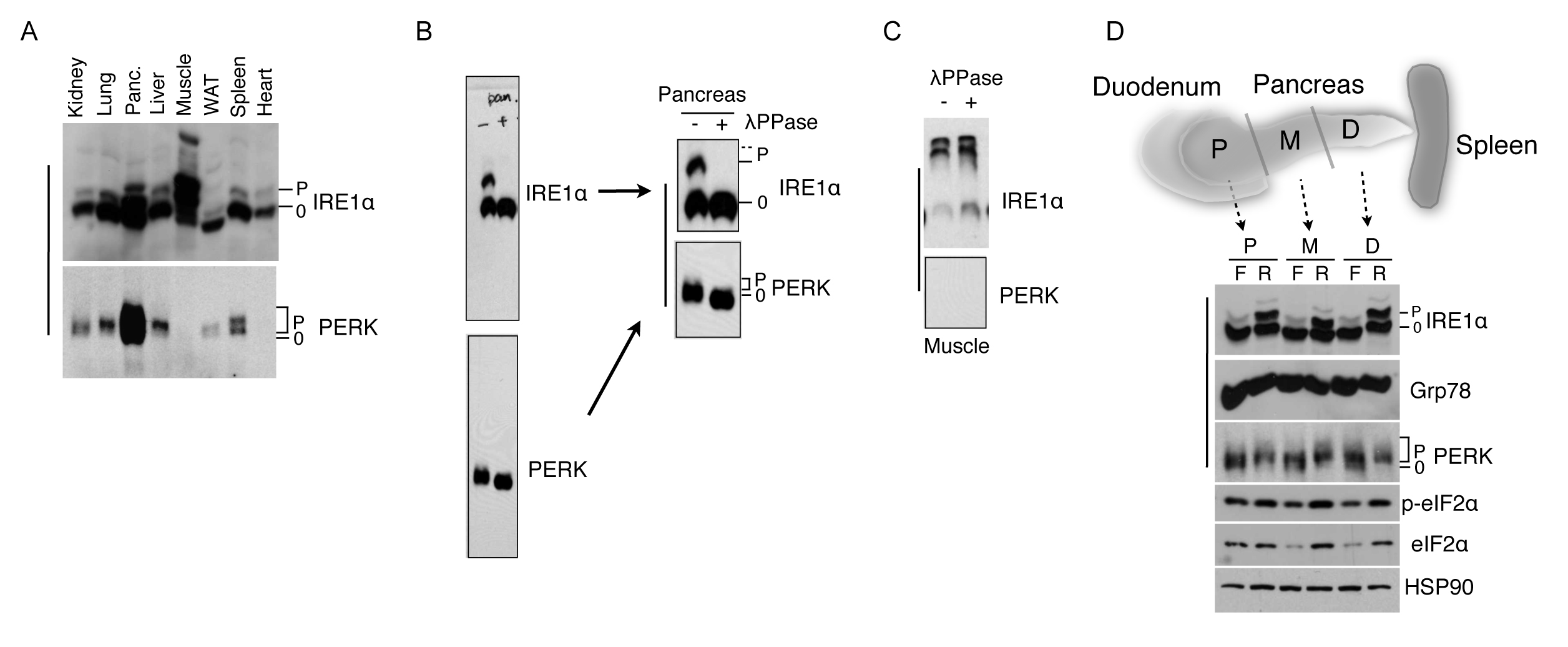

Supplement: Figure S2 — (A) Immunoblots of IRE1α (top) and PERK (bottom) in various tissues of wildtype mice under feeding conditions, an independent experiment from the one shown in Figure 3A. WAT, white adipose tissues; Panc, pancreas; Muscle, gastrocnemius. (B) Original Phos-tag whole-gel images for the data shown in Fig. 3B. Note the specificity of the antibody and the complete reverse of phosphorylation upon phosphatase treatment. (C) Immunoblots of IRE1α and PERK in muscle lysates treated with λPPase. The multiple bands of IRE1α in the muscle are not due to hyperphosphorylation and PERK protein levels are beyond detection limit. (D) Immunoblots of IRE1α and PERK in lysates extracted from different regions of the pancreas of 13-week-old wildtype mice under the 20 h-fasting (F) and 2 h-refeeding (R) conditions. The position of the pancreas is relative to the duodenum (proximal, middle or distal) - see the diagram on top. HSP90, a loading control. Phos-tag gels are indicated with a bar at the left-hand side. (0.35 MB JPG) [file pone.0011621.s002.jpg]
