# Supplementary material for: A Phos-Tag-Based Approach Reveals the Extent of Physiological Endoplasmic Reticulum Stress
Source: PLoS One. 2010 Jul 16;5(7):e11621. doi: 10.1371/journal.pone.0011621 (PMC2905412; doi:10.1371/journal.pone.0011621)
Supplement: Table S1 — Primers used in this study. (0.04 MB PDF) [file pone.0011621.s003.pdf]

	**Gene**	**Forward**	**Reverse**
**Q-PCR**	*Chop*	TATCTCATCCCCAGGAAACG	GGGCACTGACCACTCTGTTT
	Erdj4	CTTAGGTGTGCCAAAGTCTGC	GGCATCCGAGAGTGTTTCATA
	Grp78	TGTGGTACCCACCAAGAAGTC	TTCAGCTGTCACTCGGAGAAT
	Atf4	CGAGATGAGCTTCCTGAACAGC	GGAAAAGGCATCCTCCTTGC
	P58ipk	GTGGCATCCAGATAATTTCCAG	GAGTTCCAACTTCTGTGGAAGG
	L32	GAGCAACAAGAAAACCAAGCA	TGCACACAAGCCATCTACTCA
	Xbp1t, total	ACATCTTCCCATGGACTCTG	TAGGTCCTTCTGGGTAGACC
	Xbp1s, spliced	GAGTCCGCAGCAGGTG	GTGTCAGAGTCCATGGGA
